# Updating versus Exposure to Prevent Consolidation of Conditioned Fear

**DOI:** 10.1371/journal.pone.0122971

**Published:** 2015-04-22

**Authors:** Victoria Pile, Thorsten Barnhofer, Jennifer Wild

**Affiliations:** 1 Department of Psychology, Institute of Psychiatry, King’s College London, London, United Kingdom; 2 Department of Experimental Psychology, University of Oxford, Oxford, United Kingdom; Macquarie University, AUSTRALIA

## Abstract

Targeting the consolidation of fear memories following trauma may offer a promising method for preventing the development of flashbacks and other unwanted re-experiencing symptoms that characterise Posttraumatic Stress Disorder (PTSD). Research has demonstrated that performing visuo-spatial tasks after analogue trauma can block the consolidation of fear memory and reduce the frequency of flashbacks. However, no research has yet used verbal techniques to alter memories during the consolidation window. This is surprising given that the most effective treatments for PTSD are verbally-based with exposure therapy and trauma-focused cognitive behavioural therapy gaining the most evidence of efficacy. Psychological therapies aim to reduce the conditioned fear response, which is in keeping with the preliminary finding that an increased propensity for fear conditioning may be a vulnerability factor for PTSD. Our research had two aims. We investigated the degree to which individual differences in fear conditioning predict the development of PTSD symptoms. We also compared the preventative effects of two clinically informed psychological techniques administered during the consolidation window: *exposure* to the trauma memory and *updating* the meaning of the trauma. 115 healthy participants underwent a fear conditioning paradigm in which traumatic film stimuli (unconditioned stimuli) were paired with neutral stimuli (conditioned stimuli). Participants were randomly allocated to an updating, exposure or control group to compare the effects on the conditioned fear response and on PTSD symptomatology. The results showed that stronger conditioned responses at acquisition significantly predicted the development of PTSD symptoms. The updating group, who verbally devalued the unconditioned stimulus within the consolidation window, experienced significantly lower levels of PTSD symptoms during follow-up than the exposure and control groups. These findings are consistent with clinical interventions for chronic PTSD and have important implications for identifying those at risk as well as for designing novel early interventions to prevent the development of PTSD.

## Introduction

The majority of people (74% of women and 81% of men) will experience a traumatic event in their lifetime [1,2]; almost all of these people will initially develop post-traumatic stress symptoms [3] and most will recover over time [4,5]. However, 9% will go on to develop post-traumatic stress disorder (PTSD) [2]. The three most common traumas for men and women are the violent death of a friend or family member, witnessing severe injury or death and being involved in a serious motor vehicle accident [1]. PTSD is a distressing and debilitating condition which has serious long-term health implications for the individual, such as increased risk for cardiovascular disease [6,7], diabetes [8], Alzheimer’s disease [9] and early death [6] and cost implications for society, such as losses in work productivity. Re-experiencing symptoms are the hallmark feature of PTSD and include involuntary highly emotive and intensely distressing intrusions about the trauma. Meta-analyses indicate that peri-traumatic processes (occurring at or near the time of the trauma) and post-traumatic processes might be particularly important in understanding who develops PTSD and why [10,11].

There is a considerable body of research that has elucidated the memory processes involved in the development and maintenance of PTSD, including research on fear conditioning and memory consolidation [12], yet there have been surprisingly few attempts to investigate these processes in the context of prevention. This is important since there are currently no established interventions to prevent development of the disorder following exposure to trauma.

### Conditioning theory and PTSD

Conditioning theory provides a compelling account of how re-experiencing symptoms may develop [12]. Fear conditioning is a process in which a neutral stimulus (conditioned stimulus; CS e.g. a tone) gains the ability to evoke fear following repeated pairing with an aversive stimulus (unconditioned stimulus; US, e.g. a shock). This is because the CS+ (CS paired with the US) becomes a signal for US onset by activating the memory representation of the US. In general, fear conditioning is an adaptive and highly important form of learning. However, it may also be at the heart of the pathogenic mechanisms leading to anxiety disorders and has been strongly implicated in theories for anxiety disorders [13,14].

Conditioning theories offer a promising explanation of some of the central features of PTSD including why previously neutral stimuli are capable of provoking physiological and emotional arousal. Consistent with conditioning theory, evidence suggests that people with PTSD show greater physiological reactivity to trauma-related cues following trauma exposure [15], PTSD severity moderates responsively to these cues [16–18] and changes in psychophysiology can predict development and persistence of PTSD as well as treatment outcome [19–21]. Increased acquisition of fear learning (i.e. larger conditioned responding to the CS+) has been demonstrated in people with anxiety disorders [22] and there is preliminary evidence that reduced extinction learning may be a pre-trauma vulnerability factor for PTSD development [23]. A large prospective study illustrated that reduced extinction learning in soldiers before deployment to Afghanistan predicted PTSD symptom severity more strongly than neuroticism, pre-deployment stress and exposure to stressors on deployment [24].

Individual differences in fear conditioning have therefore been proposed as a predictor of PTSD but further prospective studies are needed to assess causation and exclude contamination with current post-traumatic stress. Prospective longitudinal studies have mostly been confined to at-risk occupational groups and it is unclear how far these findings can be generalized. Therefore, experimental studies are essential to build a better understanding of the causal relationship between risk factors and PTSD development. The first aim of this study was to investigate whether increased acquisition of conditioned responding predicted the development of analogue PTSD symptoms.

Modifying the conditioned fear response is a central aim in therapies for PTSD with extinction proposed as a method to achieve this. However, extinction is not permanent and re-emergence of the original fear memory can occur. Re-emergence is commonly thought to happen under three general conditions: *renewal* occurring when the CS+ is presented outside of the extinction context [25], *reinstatement* occurring when the US is administered unexpectedly and *spontaneous recovery* occurring when a substantial amount of time has passed [26]. These processes imply that extinction involves the creation of new memories (inhibitory stimulus association CS+/noUS) that compete with the original fear memory (CS+/US) rather than changing the memory directly [27,28]. An alternative to extinction, US devaluation, may offer a more fundamental way to reduce the conditioned fear response and prevent it from returning [29].

US devaluation involves changing the mental representation of the US, for example through information or experience with the US. Conditioning theory suggests that the CS+ does not directly produce a conditioned response (CR) but rather triggers a representation of the US and that the evaluation of this US mediates the CR [30]. Therefore, the strength of the CR depends on the evaluation of the US and so altering this evaluation will modulate the CR’s strength, independent of the CS/US contingency [30]. For example, if rats are habituated to the US, following CS/US pairing, then they show less fear to the CS+ compared to controls [31]. Devaluation can occur even without direct exposure to the US and results in changes to the CR when the CS+ is presented [32,33]. Effects in the opposite direction have also been documented, for example mild PTSD symptoms might become more severe if the person has reason to re-evaluate the danger of the original trauma (e.g. they found out that the person who assaulted them was a convicted murderer) [34]. The advantage of US devaluation, compared to extinction, is that it acts directly on the US representation and so may be less vulnerable to the return of fear and may more easily generalise.

Imagery rescripting, a therapeutic technique, has been proposed to act through US devaluation [29] and has been successfully applied to treating a number of anxiety disorders [35–37], including PTSD [38]. The technique involves activation of the distressing memory and then updating/rescripting of the memory with neutral or positive information. Two recent analogue studies illustrated that adding imagery rescripting to extinction training could reduce intrusion frequency [39] and the conditioned fear response at renewal as measured by US expectancy ratings but not skin conductance response (SCR) [33]. Therefore, US devaluation offers a promising technique to optimise early interventions but requires further investigation.

### Disrupting the consolidation of fear memories

When fearful memories are initially formed they appear labile and vulnerable to change but steadily become less so as they are consolidated via protein synthesis [40,41]. Invasive pharmacological manipulations have been shown to successfully disrupt consolidation but their use in humans is problematic (e.g. toxicity). Encouragingly, it has been illustrated that a non-invasive extinction procedure (i.e. not requiring pharmacological manipulation) within either the consolidation or reconsolidation window can be used to re-write the fear memory and so prevent the return of fear in rats [42,43] and humans [44,45]. Little or no reinstatement, renewal or spontaneous recovery was seen if extinction occurred shortly after conditioning, compared to moderate to strong return of fear when extinction occurred twenty-four hours or more later. The implication is that during consolidation or reconsolidation, the CS/US trace is labile and can be re-written to include the CS/noUS learning before storage. It is unclear whether extinction learning during consolidation/reconsolidation leads to the ‘unlearning’ or weakening of the stored memory trace (or access to it) and/or learning a new interpretation of the CS. It is frequently proposed that the adaptive function of reconsolidation is to enable the updating of old memories with new information [44,46].

Targeting consolidation may offer a mechanism to re-write fear memories and protect against the long-term psychological and physiological effects of traumatic fear memories. Recently, promising results have arisen from studies using visuo-spatial tasks to block the consolidation of fear memory [47]. However, no research has yet used verbal techniques to alter memories during the consolidation window. This is surprising given that the most effective treatments of chronic PTSD are verbally-based and include a particular focus on the meaning of events. Leading cognitive theories suggest that re-experiencing symptoms and intrusions are due to the fear memory lacking sufficient integration with other autobiographical memories, a deficit that may arise as a consequence of shifts in information processing under high stress [48,49].

### Therapeutic tools for early intervention

Cognitive behavioural techniques administered to people with acute stress disorder (ASD) in the first month after trauma have been shown to reduce rates of PTSD and anxiety [50,51] and are considered as the most effective early intervention so far [52]. The relative effectiveness of exposure to the trauma memory versus updating (a technique that combines exposure to the memory and cognitive restructuring) and whether these utilise different therapeutic mechanisms is contentious. Studies that have compared exposure and cognitive therapy at early intervention have shown mixed results: one illustrated comparable results in reducing PTSD [53] whilst another found that those receiving exposure were more likely to be in full remission at follow-up and achieve higher functioning than those receiving cognitive restructuring [54]. Some researchers suggest that exposure may be the critical component in CBT to prevent ASD developing into PTSD [51]. How similar the active treatment mechanism for these two techniques are, is debated and it might be that their relative effectiveness changes depending on PTSD chronicity and severity.

Therefore, the second aim of the current research was to compare common therapeutic techniques in order to inform the development of more effective interventions for early prevention. This was investigated by using trauma films to induce fear memories in healthy volunteers. The trauma film paradigm offers an ecologically valid trauma analogue to investigate causal factors in PTSD development. Previous research using this paradigm has shown that changes in appraisal style [55] leads to fewer intrusions consistent with the assumption that verbal strategies to update the meaning of the US may serve as an effective strategy to prevent re-experiencing symptoms. In order to compare the effects of the different techniques, we used a US devaluation paradigm with a range of measures, using self-reports as well as a physiological measure, to capture the complexity of PTSD. Participants underwent a fear conditioning paradigm in which traumatic film stimuli (US) were paired with neutral stimuli (CS) in the acquisition phase, and participants were then allocated to one of three groups. The groups were designed to build on one another in an additive fashion: no intervention (control group), further exposure to the trauma films (exposure group) and further exposure to the films plus additional information aimed at updating the meaning of the trauma (update group).

### Hypotheses

As this is this is the first study to use trauma film stimuli as the US, it was important to first establish whether trauma film stimuli could be used to induce conditioned fear as measured by SCR and distress ratings. The study then investigated the two main hypotheses. First, that participants who have a smaller conditioned fear response following acquisition will experience fewer intrusions, be less distressed by them and report fewer PTSD symptoms in the week following the trauma paradigm when trait anxiety is controlled for. As pre-trauma anxiety is associated with the development of PTSD [4,11] and people with higher trait anxiety have been shown to acquire aversive conditioning more strongly and more rapidly [56,57], it is important to control for trait anxiety when investigating this relationship. Second, that participants in the update group will (a) have the largest reduction in physiological responding and subjective distress ratings following US devaluation compared to those in the exposure and control groups and will (b) experience fewer intrusions, be less distressed by them and have fewer PTSD symptoms in the week following the trauma paradigm than the exposure or control groups.

## Methods

### Participants

We recruited healthy participants by poster and email advertisements. Exclusion criteria included being less than 18 years of age, having completed similar trauma studies, working in a hospital emergency department and having a clinically significant mental health problem as assessed by standardised self-report measures of anxiety, depression and PTSD. Twenty-five participants were excluded due to having clinically significant mental health problems and six participants were excluded for having previously completed a similar study or for having experience in an emergency department. One hundred and fifteen participants met inclusion criteria and completed the experimental task: 28 males and 87 females with a mean age of 26.72 (SD = 7.84; range 18–56). Of these, two participants did not complete the follow-up questionnaires leaving a sample of 113 participants for analyses involving follow-up data. All participants gave informed consent and were paid for their participation. The Psychiatry, Nursing and Midwifery Research Ethics Committee at Kings College London gave approval for the study. All participants provided written informed consent prior to their participation in the study.

### Procedure

**Fig 1:**
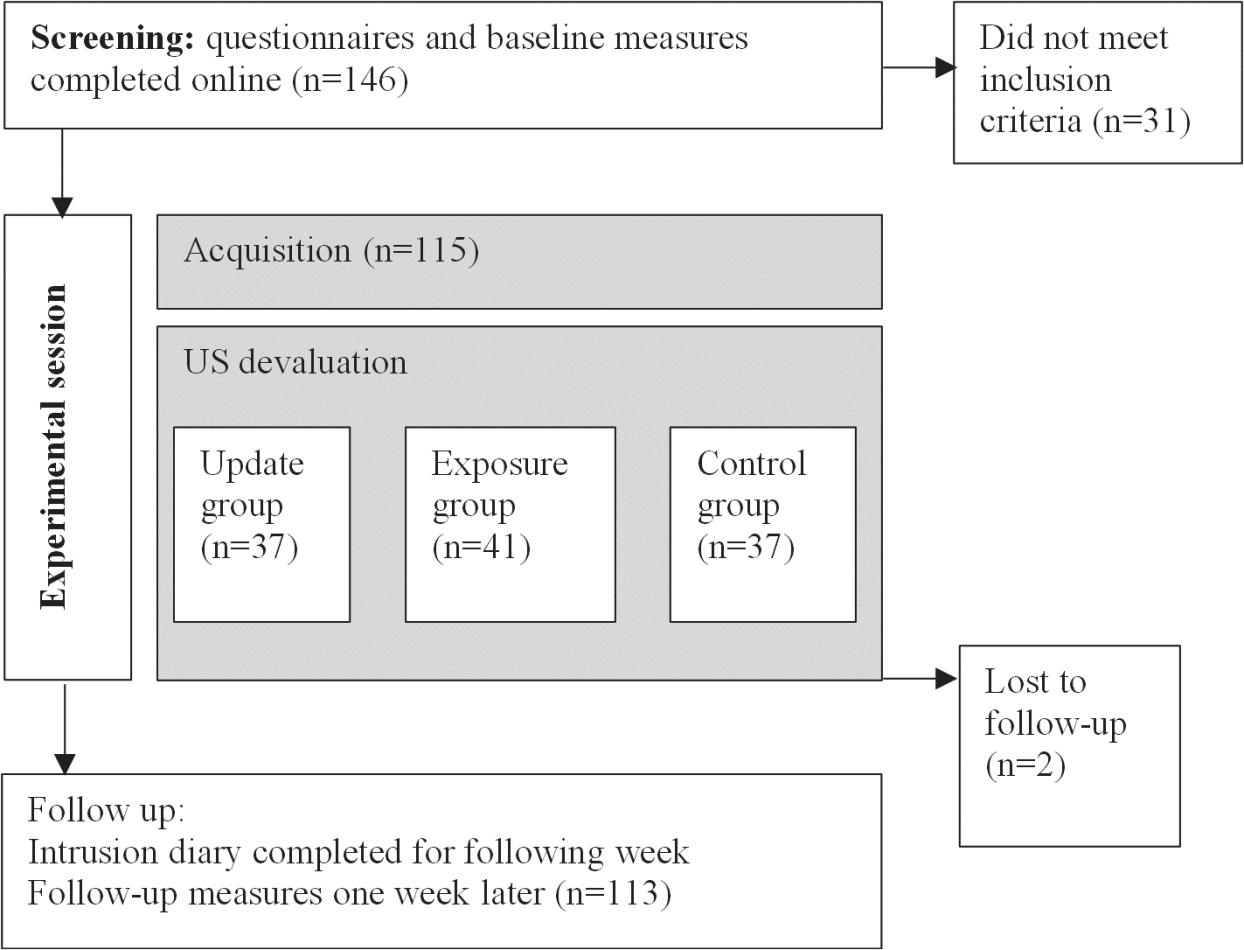
Summary of procedure.

We used an experimental between-subjects design with three main stages: a screening stage, experimental session and a one-week follow-up period. After having time to read and consider the information sheet, participants were asked to complete the baseline measures and screening questionnaires on-line before attending the session. The experimental session comprised two consecutive phases: (1) acquisition and (2) US devaluation (Fig 1). During acquisition, all participants underwent a Pavlovian discrimination fear conditioning paradigm. Fear conditioning was measured using SCR amplitude and subjective distress ratings. Participants then took part in one of three US devaluation conditions, to which they had been allocated based on a predetermined sequence produced by random number generation. In the week following the experimental phase, participants were asked to complete an intrusion diary to record any spontaneously occurring memories that they experienced related to the film clips and to complete the follow-up questionnaires.

#### Experimental session

Subjects were instructed to pay attention to the computer screen and to try to work out the meaning of the CS+ and CS-. Trauma film stimuli were used as the US, a circle (0.5 inches in diameter) as the CS+ and a square (0.7 inches in length) as the CS-. At pre-determined points during the task, the CS+ and CS- appeared in the right hand corner of the screen for two seconds. The participant’s physiological and subjective responses to the CS+ and CS- were measured before and after each phase.

All participants took part in the acquisition phase. During acquisition, participants watched a series of six short film clips with introductory narratives and the CS+s embedded within them. The films contained real-life footage of humans and animals in distress. Each film contained an introductory voice-over which gave a narrative and context for the film (see S1 Appendix), as suggested by previous research [58]. The CS+ was inserted into the films to signal the most distressing part of the film. The length of the film clips ranged from 76 to 150 seconds, with a mean length of 108.5 seconds (SD 33.09). After watching the six trauma films, participants viewed an *intensive presentation phase* in which the image and sound bite from the parts of the films previously paired with the CS+ (the worst moments) were presented again with the CS+. In this phase, the CS- was paired with neutral images. The CS+ was presented 10 times and the CS- presented 6 times during this phase. The US (an image and sound bite of the worst moment from each of the trauma films) was displayed for 3 seconds.

In the US devaluation phase, participants were randomly allocated to one of three US devaluation groups: (1) update group (exposure plus verbal update): participants viewed the same films again but the introductory passage was elaborated to contain additional information about what happened to the protagonists in the films, (2) exposure group: the trauma films with the introductory passages were viewed again by participants, (3) control: participants viewed non-traumatic films of related content. Reinforced CSs were not presented for any of the groups in this phase.

The control group viewed films whose content was similar to the trauma films and would be commonly encountered by the participant, i.e. car journeys. In order to ensure that the control films alone did not provoke significant levels of distress and fear and that the content was rated as neutral, they were viewed by six pilot participants. The participants were asked to rate the control films for feelings of distress and fear (measured on a scale of 0 being not at all distressing/fear provoking to 100 being extremely distressing/fear provoking) and rate the valence of the films (with 0 being very positive and 100 being very negative). The films were rated as not producing significant distress (mean 2.50; SD 4.18) or fear (mean 2.50; SD 4.18) and their valence was rated as neutral (mean 48.67; SD 2.16). No intrusions related to the control films were reported by pilot participants.

### Psychophysiological Assessment

Skin conductance response (SCR) signals were amplified with a BrainVision Quickamp with 22-bit A/D conversion and a resolution of 71.5 nV (range7150 mV) and digitised at a rate of 125 Hz. SCR was recorded through an auxiliary channel.

Electrodermal activity (EDA) was measured as skin conductance response (SCR) in constant voltage technique using the GSR module produced by Brain Products, which applied a constant voltage of 0.5 V. Sintered silver-silver chloride (Ag-AgCl) cup electrodes were attached to volar surfaces of the medial phalanges of the second and third finger of the non-dominant hand, using MedCaT skin conductance electrode paste (0.05M NaCL saturation) as electrolyte. The electrodes were metal discs set in cylindrical plastic cases that were filled with electrode gel and fixed to the skin using double-sided adhesive circular collars. A bandpass filter of 0.0Hz to 2.00Hz was applied.

EDA data were segmented into 2 second epochs (beginning 2 seconds pre-stimulus) and 8 second epochs (beginning post-stimulus). SCR amplitude was calculated as the difference between the mean SCR level for the 2 seconds preceding stimulus onset and the highest SCR value during the 8 seconds following stimulus onset, as has been done in recent studies [59]. A minimum response criterion of 0.02μs was applied in this study. Participants who did not show an SCR above this minimum on the acquisition trials were scored as zero and included in the analysis, consistent with recent studies [59–61].

### Demographic and Screening Measures

Participants were asked to complete screening measures and a questionnaire to assess demographic factors before attending the experimental session. This was to exclude participants with clinically significant levels of mental health problems and to match the groups according to demographic factors, levels of anxiety, PTSD and depression and trauma history. The measures that were used to screen for clinically significant mental health problems are those routinely used in primary care in the UK.

#### The Patient Health Questionnaire—nine items (PHQ-9 [62,63])

The PHQ-9 is a widely used, reliable and well-validated nine item self-report measure of depression in the general population [63]. On the PHQ-9, participants are required to rate how often they have experienced symptoms of depression in the last 7 days on a scale of 0 (not at all) to 3 (nearly every day). Scores range from 0 to 27 with higher scores indicating more severe depression. A cut-off score of 10 or above (suggests moderate depression) is used in primary mental health services in the UK (http://www.iapt.nhs.uk) and was used in this study.

#### The Generalised Anxiety Disorder Assessment—seven items (GAD-7, [64])

The GAD-7 was used to screen participants for clinically significant levels of anxiety and to match groups on anxiety symptomatology. The GAD-7 was originally developed as a screening tool for generalised anxiety disorder but has been shown to also screen for other common anxiety disorders included panic disorder, PTSD and social anxiety disorder [65]. The GAD-7 is used extensively in primary care as a screening tool for anxiety with a recommended a clinical cut-off of 8 (http://www.iapt.nhs.uk) which was used in this study. The GAD-7 has been shown to be a reliable and valid measure of anxiety [64].

#### The Trauma Screener (unpublished)

The Trauma Screener is a self-report checklist of traumatic events. It was administered to match the groups for prior trauma exposure and to identify the participant’s most stressful life event for them to reference when completing the baseline IES-R. The Trauma Screener has been used in previous studies [66] and was based on the trauma checklist from the Clinician-Administered Post-Traumatic Scale [67].

#### The Impact of Events Scale-Revised (IES-R [68])

The IES-R is a twenty-two item measure that asks participants to rate the distress caused by their symptoms from 0 (not at all) to 4 (extremely) over the last 7 days with respect to a traumatic event that they have experienced. Higher scores indicate greater PTSD symptom severity with a total score of 88. The trauma screener was used to identify the index trauma for completion of the IES-R at baseline and a clinical cut-off of 33 was used as recommended by previous research [69].

### Other Measures

#### Spielberger State-Trait Anxiety Inventory-Trait version (STAI-T [70])

The STAI-T is a twenty item self-report measure of trait anxiety i.e. a tendency to perceive situations as threatening and to increase state anxiety in response to them. The STAI is frequently used in research and clinical practice with internal consistency coefficients ranging from 0.86 to 0.95 and test-retest reliability coefficients ranging. 65 to. 86 [70]. Studies have also demonstrated construct and concurrent validity [71]. Participants were asked to complete the STAI-T before taking part in the experimental session. The STAI-T was used as predictor of analogue PSTD symptoms and not as a screening tool.

#### Subjective ratings of distress (unpublished)

Visual analogue scales were used to assess subjective distress to the conditioned and unconditioned stimuli. Participants were asked to rate how distressing they found the stimuli anchored with 0 (not at all) to 100 (extremely distressing) at 4 different time-points in the films. Previous studies have indicated that a general term for emotion (e.g. distress) is a reliable index for gauging shifts in emotion in response to experimental manipulation and subjective units of distress (SUDS) are commonly used in clinical research and clinical settings [55,72,73].

#### Intrusions diary

After viewing the films, participants were asked to keep a daily intrusion diary for one week to assess the number of intrusive memories and distress experienced. This method of assessing intrusions has been used frequently in analogue trauma paradigms [74]. Participants are asked to record the number of intrusions they experienced and their level of subjective distress (rated as 0–10) in relation to the intrusions. At follow-up, Participants were asked to rate how accurately and reliably they had completed the intrusions diary (diary compliance). Participants were asked to rate two questions (accuracy and reliability) on a 10 point likert scale from 0 (not at all) to 10 (extremely).

#### The Impact of Events Scale-Revised (IES-R [68])

At follow-up, participants were asked to complete the IES-R with reference to the trauma films. Adapting the IES or IES-R to provide a measure of responses to the trauma films has been done in previous studies [47,75,76]. The IES has been shown to correlate with frequency of intrusions [76] further indicating that it is a valid measure for assessing intrusion symptomatology in analogue studies.

### Power analysis

A power analysis was conducted using Cohen's power primer with the effect sizes based on a previous study with a similar experimental design [44]. Cohen’s power primer indicated a sample size of n = 21 per group with alpha set at 0.05 and power at 80% for a large effect. Initial piloting revealed that some participants demonstrate poor electrodermal responsivity (as measured by skin conductance response, SCR). From the piloting stage, it was estimated that approximately 25% would fail to produce a SCR above 0.02μs (which the power analysis is based on) and so the required sample size was estimated to be 84.

### Statistical analysis

A two-tailed significant level of α = 0.05 was used throughout analysis. For baseline characteristics, differences between the groups were analysed using Chi-squared analysis for categorical variables and a combination of one-way ANOVAs and their non-parametric equivalent for age, screening questionnaires, CS+ACQ (SCR and distress ratings) and SCR during the films and diary compliance.

In order to test our first hypothesis, we used a multivariate analysis of covariance (MANCOVA) to analyse the relationship between an individual’s conditioned acquisition response, trait anxiety and their intrusion frequency, intrusion distress and PTSD symptomatology whilst controlling for the effect of group allocation. This analysis was conducted with group as a categorical variable, conditioned SCR following acquisition and trait anxiety as co-variates and IES-R, intrusion frequency and distress as dependent variables. The analysis was repeated with differential subjective distress following acquisition as a predictor variable.

There were two stages of analysis to test our second hypothesis. First, to compare the changes in SCR amplitudes between the groups, an ANCOVA for analysis of repeated measures was used with SCR following US devaluation as the dependent variable, group as the fixed factor and SCR following acquisition as the covariate. This method of analysis is consistent with recommendations [77]. Only unreinforced trials of CS+ were included in the analysis in order to assess response to the CS+ alone separate from unconditioned responses to the trauma films. The data did not meet distributional assumptions of a regression and so bootstrapping was used for inference. Bootstrapping is a non-parametric, computer-intensive approach to statistical inference that gives valid standard errors, confidence intervals and p values for hypothesis tests without the normality assumption. It is based on building a sampling distribution for a statistic by resampling with replacement from the sample data. It only assumes that the sampled data provide a reasonable representation of the population from which they came and therefore do not have distributional assumptions [78]. The analysis was repeated with subjective distress following US devaluation as the dependent variable, distress following acquisition as the covariate and group as the fixed factor.

Second, a multivariate analysis of variance (MANOVA) was used to compare intrusion frequency, intrusion distress and IES-R scores for the three groups with follow-up tests using separate univariate ANOVAs and independent t-tests to examine individual group differences.

## Results

Of the 115 participants, *n* = 37 were allocated to the update group, *n* = 41 to the exposure group, and *n* = 37 to the control group. The groups were well-matched on criteria related to the hypotheses examined in this study (i.e. demographics, the screening measures, fear conditioning following acquisition, SCR during the trauma films and reliability in completing the intrusions diary). At baseline, the groups did not differ in terms of demographic variables, self-reported anxiety, depression, trauma history and PTSD symptoms (all *p* >. 10; Table 1).

**Table 1:** Baseline measures.

	Update Mean (SD) (n = 37)	Exposure Mean (SD) (n = 41)	Control Mean (SD) (n = 37)	Statistical analysis
**Age**	25.49 (8.34)	27.12 (8.86)	26.97 (7.51)	H(2) *=* 2.01, p = .904
**Sex**	27 females	32 females	28 females	χ^2^(2) = .272, *p* = .962
**STAI-T**	34.54 (8.17)	35.78 (9.44)	33.76 (9.11)	F(2,112) = .505, p = .605
**IES-R**	6.81 (8.68)	7.48 (8.69)	6.14 (8.27)	F(2,112) = .207, p = .814
**PHQ-9**	1.49 (1.98)	2.00 (2.33)	1.65 (2.37)	F(2,112) = .996, p = .373
**GAD-7**	1.57 (1.74)	1.70 (1.97)	1.35 (1.98)	F(2,112) = .848, p = .431
**Trauma screener**	2.23 (1.87)	1.83 (1.75)	2.24 (2.08)	F(2,112) = .39, p = .678

Data were log transformed prior to analysis. Untransformed values are reported.

Participants all received the same acquisition conditioning and there were no significant differences between the groups in SCR or self-reported distress to the CS+ at acquisition (CS+ACQ) (Table 2) or SCR during the worst moments in the trauma films (Table 3).

**Table 2:** Subjective distress ratings and SCR to CS+ following acquisition (means and bootstrapped standard errors).

CS+ Acquisition	Update (n = 37)	Exposure (n = 41)	Control (n = 37)	One way ANOVA
**Subjective distress rating**	53.83 (5.17)	41.34 (4.20)	42.70 (4.47)	F(2,112) = 2.23, p = .113
**SCR**	0.19 (0.053)	0.24 (0.048)	0.20 (0.050)	F(2,112) = 0.27, p = .76

**Table 3:** SCR during acquisition films (means and bootstrapped standard errors).

	Update (n = 37)	Exposure (n = 41)	Control (n = 37)	One way ANOVA
**Film 1**	0.54 (0.089)	0.45 (0.069)	0.45 (0.090)	F(2,112) = 0.37, p = .69
**Film 2**	0.33 (0.087)	0.37 (0.070)	0.37 (0.093)	F(2,112) = 0.79, p = .92
**Film 3**	0.50 (0.086)	0.61 (0.11)	0.55 (0.11)	F(2,112) = 0.25, p = .78
**Film 4**	0.11 (0.031)	0.20 (0.039)	0.13 (0.063)	F(2,112) = 0.91, p = .41
**Film 5**	0.33 (0.068)	0.36 (0.069)	0.34 (0.075)	F(2,112) = 0.034, p = .97
**Film 6**	0.24 (0.057)	0.30 (0.066)	0.25 (0.062)	F(2,112) = 0.33, p = .72

The groups did not differ in terms of their reliability and accuracy in completing the intrusions diary (Table 4).

**Table 4:** Self-reported diary compliance at follow-up.

diary compliance	Update (n = 37) Mean (SD)	Exposure (n = 41) Mean (SD)	Control (n = 36) Mean (SD)	ANOVA
**Accurate**	8.68 (1.20)	8.29 (1.23)	7.86 (1.87)	F(2,111) = 1.916, p = .152
**Reliable**	2.3 (1.94)	2.12 (1.58)	2.17 (1.65)	F(2,111) = 0.72, p = .931

Data were log transformed. Untransformed values are reported.

Fear conditioning, as measured by SCR amplitude and subjective distress ratings, occurred using trauma film stimuli as the US. There was a significant difference in response to CS+ and CS- at acquisition as measured by both SCR, t(114) = 4.49, p<0.01, r = .39, and subjective ratings of distress, t(114) = 15.49, p<0.01, r = .82 (see Table 5). In addition, the paradigm successfully elicited intrusive memories of the films with 95% of participants reporting at least one.

**Table 5:** CS+ compared to the CS- following Acquisition using SCR and subjective distress ratings (means and bootstrapped standard errors).

	CS+ Acquisition	CS Acquisition	Paired Sample T-Test
**SCR**	0.21 (0.028)	0.075 (0.017)	t(114) = 4.49, p = 0.00, r = .39
**Subjective rating (all cases)**	45.80 (2.61)	4.96 (1.13)	t(114) = 15.49, p = 0.00, r = .82

### Conditioned fear: a biomarker for PTSD

The differential conditioned fear response following acquisition was calculated as the mean SCR amplitude to the unreinforced CS+ACQ minus the SCR amplitude to the unreinforced CS-ACQ. The analysis was then repeated using ratings of subjective distress rather than SCR. A MANCOVA was conducted with group as a categorical variable, intrusion frequency, intrusion distress and IES-R as the dependent variables and differential SCR as a covariate. Analysis (*N* = 113), using Pillai’s trace, revealed a significant effect of the covariate, differential SCR, on the dependent variables, *V* = 0.12, *F*(3, 107) = 3.48, *p* <. 01, partial *η* ^2^ = .12. There was a significant effect of group after controlling for differential SCR, *V* = 0.18, *F*(6, 216) = 3.46, *p* <. 01, partial *η* ^2^ = .09. When trait anxiety (as measured by the STAI-T) was included as a covariate in the above analysis, the relationship between trait anxiety and the dependent variables was at trend level significance, *V* = 0.01, *F*(3, 106) = 2.51, *p* = .06. The significant effect of group and differential SCR on the dependent variables remained when trait anxiety was taken into account. Follow-up tests revealed that differential SCR was significantly related to intrusion frequency, *F*(1, 109) = 1.49, *p* <. 01, partial *η* ^2^ = .08, intrusion distress, *F*(1, 109) = 3.52, *p* <.01, partial *η* ^2^ = .08, and IES-R score, *F*(1, 109) = 1.15, *p* <. 01, partial *η* ^2^ = .07.

Differential subjective distress was calculated using the same method (subjective distress rating to the CS+ at acquisition minus subjective distress rating to the CS- at acquisition). Analysis, using Pillai’s trace, revealed a significant effect of differential subjective distress on the dependent variables, *V* = 0.14, *F*(3, 107) = 5.64, *p* <. 01, partial *η* ^2^ = .14 and a significant effect of group, *V* = 0.18, *F*(6, 216) = 3.62, *p* <. 01, partial *η* ^2^ = .92. Follow-up tests revealed that differential subjective distress was significantly related to intrusion frequency, *F*(1, 109) = 7.69, *p* <. 01, partial *η* ^2^ = .066 and intrusion distress, *F*(1, 109) = 14.21, *p* <.01, partial *η* ^2^ = .12.

These findings imply that participants who acquired a stronger conditioned fear response, as measured either by SCR or subjective ratings of distress, experienced more intrusions and were more distressed by them. Further to this, participants with a stronger conditioned fear response as measured by SCR experienced more PTSD symptoms following the trauma film paradigm.

### Targeting the consolidation of fear conditioning

**Fig 2:**
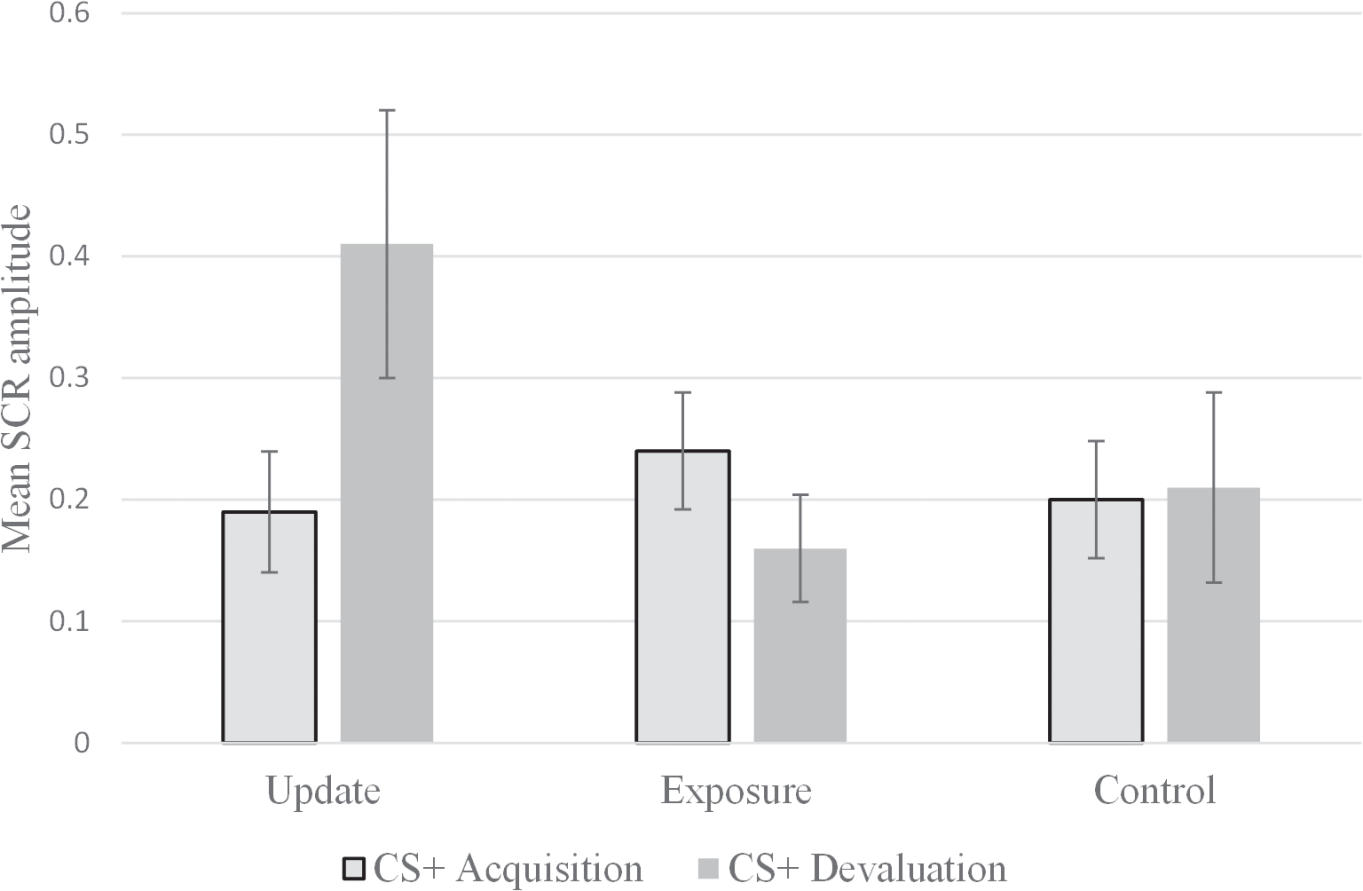
Verbally updating the trauma memory increases SCR response to CS+. A significantly larger increase in SCR to the CS+ from acquisition to US devaluation was found in the update as opposed to the exposure and control groups. The three groups were equivalent at acquisition. Error bars represent bootstrapped standard errors.

SCR amplitude to the CS+ at acquisition and at US devaluation is represented in Fig 2 (see Table 6 for descriptive statistics). Analysis with bootstrapped data revealed that there was a significant difference between the groups in SCR following devaluation after controlling for effect of SCR at acquisition, *F*(2,111) = 6.57, p<0.01, partial η^2^ = .11. SCR following acquisition was also significantly related to SCR following US devaluation, *F*(3,111) = 99.74, p<0.01, partial η^2^ = .473. Post-hoc tests revealed that the update group showed a significantly larger increase in SCR amplitude to the unreinforced CS+ following US devaluation compared to the exposure group, (mean difference: 0.31; bootstrapped confidence intervals: 0.14 to 0.48; p<0.01) and control group (mean difference: 0.21; bootstrapped confidence intervals: 0.022 to 0.41; p<0.05).

**Table 6:** SCR and distress ratings for the CS+ for each group following acquisition and devaluation (means and bootstrapped standard errors).

	Measure	CS+ Acquisition	CS+ Devaluation
**Update (n = 37)**	SCR	0.19 (0.0496)	0.41 (0.11)
	Distress ratings	53.84 (4.95)	36.54 (4.04)
**Exposure (n = 41)**	SCR	0.24 (0.048)	0.16(0.044)
	Distress ratings	41.34 (4.13)	39.70 (4.29)
**Control (n = 37)**	SCR	0.20 (0.048)	0.21 (0.078)
	Distress ratings	42.70 (4.44)	36.35 (4.49)

The same analysis was repeated with subjective distress ratings at US devaluation as the dependent variable. There was a significant main effect of group on distress ratings at US devaluation after controlling for distress ratings at acquisition, *F*(2,111) = 3.52, p<0.05, partial η^2^ = .60. Subjective ratings of distress at acquisition were also significantly related to ratings at devaluation, *F*(1,111) = 111.75, p<0.01, partial η^2^ = .50. Post-hoc tests revealed that the update group had a significantly larger drop in subjective ratings of distress to the CS+ at US devaluation than the exposure group (mean difference: -11.55; bootstrapped confidence intervals: -19.46 to -3.33; p<0.01) and the difference between the update and control group was at trend level significance (mean difference: -7.28; bootstrapped confidence intervals: -16.06 to 1.14; p<0.1).

**Fig 3:**
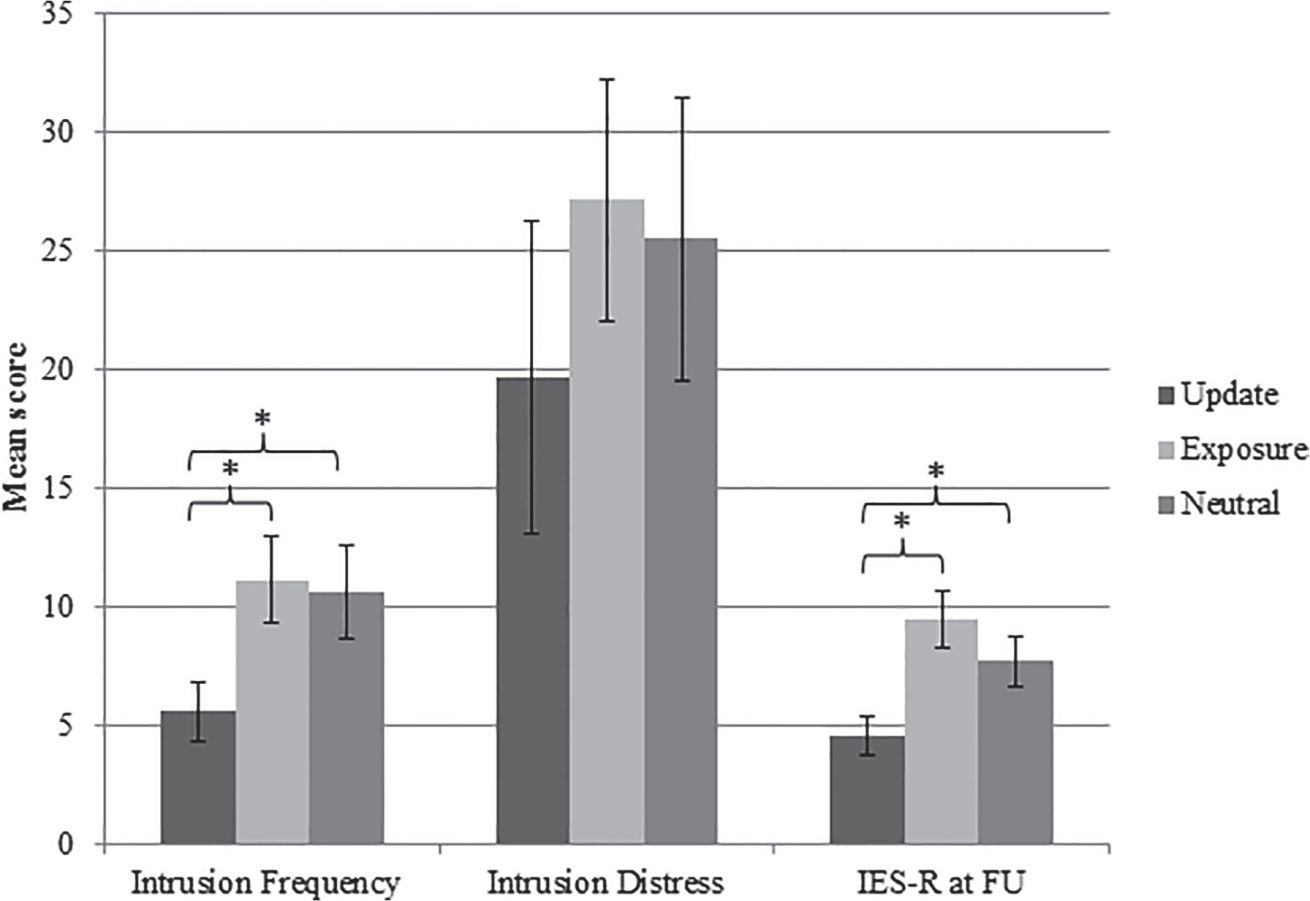
Verbally updating the trauma memory reduces intrusion frequency, distress and PTSD symptoms. Mean intrusion frequency, distress associated with the intrusions and PTSD symptom scores over the week following the experimental session for each group (update, exposure and control)**.** The update group reported significantly fewer intrusions and PTSD symptoms than the other two groups. * *p* < 0.05 (between updating and exposure groups or between updating and control groups). The difference in intrusion distress between the groups approached significance, *p* < 0.075. Error bars represent standard errors.

To investigate group differences in PTSD symptomatology, a MANOVA was conducted with intrusion frequency, intrusion distress and PTSD symptom scores as dependent variables and group as a fixed factor. Fig 3 illustrates the relative group differences (see Table 7 for descriptive statistics). This revealed, using Pillai’s trace, a significant effect of group on the dependent variables, *V* = 0.18, *F*(6, 218) = 3.50, *p* <. 01, partial *η* ^2^ = .08. Separate univariate ANOVAs were conducted on the dependent variables and showed significant differences between the groups on intrusion frequency, *F*(2, 110) = 5.99, *p* <. 01, *r* = .31, and PTSD symptom scores, *F*(2, 110) = 7.70, *p* <. 01, *r* = .35. Follow-up analysis using independent t-tests revealed that the update group reported significantly fewer intrusions and PTSD symptom scores than the exposure group (intrusion frequency: *t*(75) = -3.35, *p* <. 01, *r* = .36; PTSD symptom score: *t*(75) = -3.73, *p* <. 01, *r* = .40) or the control group (intrusion frequency: *t*(71) = -2.61, *p* <. 05, *r* = .30; PTSD symptom score: *t(*71) = -2.71, *p* <. 01, *r* = .31). These results imply that verbally devaluing the US reduces subjective distress, intrusion frequency and PTSD symptomatology but increases SCR to the CS+.

**Table 7:** Intrusion frequency, Intrusion Distress and IES-R for each group.

	Update mean (SD)	Exposure mean (SD)	Control mean (SD)
**Intrusion Frequency**	5.60 (7.66)	11.15 (11.45)	10.64 (11.86)
**Intrusion Distress**	19.68 (40.25)	27.15 (32.18)	25.50 (35.63)
**IES-R at FU**	4.54 (4.94)	9.47(7.73)	7.72 (6.28)

Data were log transformed. Untransformed values are reported.

## Discussion

Prospective experimental studies are needed to better understand causal factors in PTSD and to develop early interventions but it is clearly unethical to induce real trauma in people. Therefore, the well-established trauma-film paradigm was used to investigate causal factors in analogue PTSD symptom development and the relative effectiveness of clinically-informed psychological techniques. Trauma films have not previously been used as a US with most conditioning studies using electric shocks as the US [44,60]. Using trauma film stimuli as the US has advantages of being a better analogue of real-life trauma, being more readily experimentally manipulated (e.g. they can be devalued cognitively) and allowing the concurrent investigation of conditioned fear and PTSD symptomatology. This study demonstrated that trauma film stimuli can be used as the US to induce conditioned fear as measured by SCR and distress ratings. The trauma paradigm was effective in inducing intrusive memories of the films with 95% of participants reporting at least one intrusion. Therefore, this current study suggests that trauma film and conditioned fear paradigms can be effectively combined.

In terms of hypothesis one, participants who acquired a stronger conditioned fear response experienced more intrusions, were more distressed by them and experienced more PTSD symptoms following the trauma film paradigm. Differential SCR at acquisition predicted all three of the outcome variables whilst differential subjective ratings of distress predicted number of intrusions and distress in response to the intrusions. This is consistent with conditioning theories of PTSD which highlight the role of CS-US contingencies in the development of PTSD [26,56] and previous research demonstrating that people with anxiety disorders acquire fear conditioning more strongly than those without [22]. The relationship between enhanced fear conditioning and anxiety disorders may operate at a number of stages e.g. a stronger association may mean that the fear response is more readily triggered to cues, triggered to more loosely associated cues, is more difficult to extinguish or that the return of fear is more likely following extinction. This current finding is consistent with the proposal that individual differences in the ease of acquisition can explain why some people develop anxiety disorders and some people do not [79]. To our knowledge, this is the first study to combine a conditioning paradigm with the trauma film paradigm to enable a prospective analogue design investigating how individual differences in fear conditioning impact on PTSD symptom development. Therefore, these results indicate that, in the non-clinical population, conditioned acquisition response is a better predictor of intrusion frequency, intrusion distress and PTSD symptoms than trait anxiety.

In terms of hypothesis two, the results indicate that verbally devaluing the US reduces subjective distress, intrusion frequency and PTSD symptomatology but increases SCR to the CS+. This finding is consistent with SCR reflecting an orienting attentional response [80]. There is evidence to suggest that successful therapy results in an increase in attentional allocation to threat cues to enable the re-appraisal of the threat cue [81]. Early elevations in psychophysiology during imaginal flooding have been shown to predict improvements in intrusions [82]. Therefore, the larger SCR may represent increased fear-related arousal in the update group compared to the exposure or control groups, which is likely to be due to reduced attentional avoidance to the CS+, and, from the above perspective, corresponds well with the subsequent fall in subjective ratings of distress and the reduced number of analogue PTSD symptoms. New information and verbally enhanced representation of the trauma films may initially prevent a reduction of the conditioned fear response in the update group. Yet, in therapy, emotional arousal is considered to be important for therapeutic change [83] and the construction of new meaning is needed for lasting changes [84]. Therefore, the update condition may have facilitated higher emotional arousal (i.e. SCR) alongside new information leading to fewer intrusions and PTSD symptoms at follow-up.

Theoretically, the reduction of analogue PTSD symptoms in the updating group provides support for the idea that altering negative appraisals of the film content and enhancing verbal-conceptual processing of the trauma memory can reduce development of PTSD symptoms. This is consistent with cognitive models of PTSD which highlight the role of negative appraisals of the trauma [48] and the disorganised nature of the trauma memory in the development of PTSD symptoms [48,85]. These results also support the idea that the most effective treatments for PTSD are those that pay attention to the trauma memory and its meaning [86].

There are other possible explanations for the reduction in analogue PTSD symptoms in the update group yet these appear less plausible. First, the update group may have had a higher working memory (WM) capacity load than the other two groups. However, working memory demands were well matched in the groups and a previous study with a similar design concluded that these groups would have similar WM demands [39]. Second, the update group may have had a more positive mood than the other groups when finishing the task, as low mood is linked to greater intrusion frequency [87]. However, any impact on intrusion frequency from changes in mood would be short-lived (less than 24 hours) as illustrated by studies aiming to induce low mood [88,89] and participants in this study experienced intrusions over the entire week.

### Limitations and future directions

There are several limitations to this research. One limitation is the degree to which the findings can generalize to people who are exposed to real-life trauma and who develop PTSD since the study used non-clinical participants with films as the analogue trauma. In therapy, exposure and updating techniques are more complex than the analogue interventions used in this study. This study externally generated the updated meaning whilst, in therapy, time would be spent generating a subjective meaning with the patient and the techniques would be part of a much longer individually formulated intervention. However, the experimental interventions were generated based on those used in evidence-based therapy and the aims of both experimental techniques were in keeping with aims of evidence based therapies. The non-specific nature of SCR is another limitation and this study would have been enhanced by having additional indexes of fear conditioning, such as startle responses [60]. A further limitation is that the CS+ and CS- were not counterbalanced in this study, however as response to the CS+ following acquisition was used as a covariate, this is unlikely to impact on the findings.

As this study appears to be the first to have combined conditioning and trauma film paradigms, the results require replication. Further studies investigating whether increased conditioned fear acquisition predicts the development of PTSD in experimental settings, in at-risk groups prior to trauma exposure and in settings targeting people shortly after trauma exposure (e.g. hospital emergency departments) would illustrate whether this could be a reliable index for identifying people vulnerable to PTSD development. It would be useful to expand on pilot studies investigating whether acquisition response is associated with treatment outcome [90]. For example, it might be predicted that those who show a stronger acquisition response are more likely to develop PTSD and also more likely to derive therapeutic gain from early intervention.

Future studies could add control groups to elucidate the mechanisms that may be reducing intrusions and PTSD symptomatology in the update group. Cognitive models of PTSD [48] would predict that targeting both the disorganized nature of the trauma memory and the negative appraisals of the trauma are helpful in reducing PTSD symptoms. It would be interesting to attempt to experimentally separate these processes to establish their relative effects. For example, it may be that the update group experienced fewer intrusions due to changing the meaning of the films or that the addition of new *verbal* information disrupted consolidation or it is the combination that has therapeutic benefit. A control group that receives additional neutral verbal information to enhance verbal processing without changing meaning compared to a group that changes the meaning of the films would further understanding. There are other control groups that could be added to further tease apart the mechanisms and aid the development of effective early intervention, including visually compared to verbally updating the meaning of the trauma films and receiving the verbal update alone without exposure.

It would be important to investigate whether varying the time between acquisition and US devaluation/extinction has an impact on treatment effect. Some studies have included a thirty minute delay between trauma films and the intervention to mirror average waiting times in emergency departments [47]. Based on previous research, it is assumed that an intervention taking place in the consolidation window (approximately 6 hours) may be more effective in re-writing the trauma memory than one administered outside it [44,47]. However, further investigation is warranted into whether effectiveness changes both within this six-hour window and outside it.

If the results are replicated and experimental effect is established, a study using cognitive behavioural techniques as early interventions in real-life settings (such as hospital emergency departments) would be warranted to establish whether updating techniques can limit the development of PTSD. It is important in this context to see that the verbal updating used in the current study differs significantly from debriefing techniques that advertently or inadvertently elaborate trauma memories, and instead is aimed at containing meaning and changing it in a more benign direction, akin to the mechanisms of memory re-scripting techniques, which have proven highly effective in the treatment of anxiety disorders [91,92]. For example, debriefing occurs in group or individual format and typically includes asking participants to speak about their experience and how it has affected them, focusing on normalising symptoms and brainstorming coping strategies. In contrast, if the verbal updating used in the current study was applied clinically, it would focus on exposure to the trauma memory through reliving techniques, identifying the meaning of the event for the person and using cognitive restructuring techniques to verbally elaborate the memory past the worst moments.

## Conclusions

Consistent with conditioning theories of PTSD [26,56], we have demonstrated that individual differences in acquiring CS/US contingencies predict subsequent PTSD symptoms. Further to this, as predicted by cognitive models [48,49], devaluing the trauma memory with a verbal intervention successfully reduced distress associated with the conditioned stimulus and prevented development of symptoms. This supports the idea that the most effective treatments for PTSD are those focusing on the trauma memory and its meaning [86]. Verbally enhancing the trauma memory is proposed to reduce intrusion frequency via increased integration and contextualisation of the memory allowing better top-down control. Previous research has suggested that exposure to the trauma memory may be the key ingredient for therapeutic change [52,93]. This study provides initial evidence that changing the meaning of the US may devalue the US more effectively than exposure alone. The increase in SCR amplitude in the update group is consistent with treatment studies illustrating initial increases in psychophysiological responding predicting improvements in intrusions [82]. Successful therapy may initially lead to an increase in attentional allocation to threat cues to allow re-appraisal of the threat cue [81] and this would be reflected in a larger SCR.

The current results have significant implications for the treatment of PTSD [94], especially early intervention, and identifying those at risk for developing PTSD. Blockade of reconsolidation and extinction are the paradigms frequently used to reduce conditioned fear. Both are limited in their efficacy as blockade often requires the use of toxic drugs and conditioned fear has been shown to return following extinction. Verbally devaluing the US representation offers a safe non-invasive intervention grounded in evidence from clinical outcome studies and conditioning theory.

## Supporting Information

S1 Appendix Trauma film narratives. (DOCX)
