# Supplementary material for: Updating versus Exposure to Prevent Consolidation of Conditioned Fear
Source: PLoS One. 2015 Apr 22;10(4):e0122971. doi: 10.1371/journal.pone.0122971 (PMC4406712; doi:10.1371/journal.pone.0122971)
Supplement: S1 Appendix — (DOCX) [file pone.0122971.s001.docx]

**Supporting Information**

**Appendix S1. Trauma film narratives.**

**Appendix 1: Trauma film narratives**

**Introductory Narratives**

1. A hard-working married couple are going away for the weekend. It is their 25th Wedding Anniversary. The husband has planned a surprise country break for his wife. They are driving to their hotel on the same road as many lorries.
2. An elderly man’s wife dies after a long battle with cancer. He is distraught. He is depressed and disorientated and finds himself on the motorway.
3. In some parts of Spain, bullfighting still takes place. Sharp darts are fired into the bull’s back, and it is let loose into the streets. The sharp darts draw blood and are painful, hitting key nerves close to the animal’s spine, causing the bull to become frightened and to fight for his survival.
4. On a Monday morning at 8:30am, a disastrous pile up is caused on the motorway by a fog zone coming up within a few seconds. As a consequence, 200 cars crash into each other and leave a roadway of 200 yards covered by car wreckage. When people get out of their cars to understand what had happened, a lorry approaches too fast and crashes into the pile up. The truck pushes five cars for a distance of 50 yards compressing them into a small metal ball that is finally stopped by hitting a travel bus. Some of the cars catch fire and burn out before the passengers can free themselves. On the top of the ball of compressed cars, a mother of two is still alive. It is four hours before she is rescued. Her two children are buried underneath the car and she does not know if they are dead or alive.

1. An 18 year old and his friend are celebrating the success of their A-levels. They are driving home from a party at their school in their parents' car.
2. A young woman is 6 months pregnant. She and a friend decide to go out for lunch. Her friend picks her up and they drive to a restaurant in the next town.

**Updating**

1. A hard-working married couple are going away for the weekend. It is their 25th wedding anniversary. The husband has planned a surprise country break for his wife. They are driving to their hotel on the same road as many lorries. A lorry spins out of control and collides with two cars on the motorway causing an accident with a number of cars. The husband and wife survive the accident with many injuries but they see many motorists who do not survive. The husband and wife are shaken up and drive home to their families after the accident rather than to their hotel. They want to be close to loved ones and to let them know how much they care about them.
2. An elderly man’s wife dies after a long battle with cancer. He is distraught. He is depressed and disorientated and finds himself on the motorway. The elderly man takes his life. He dies instantly and painlessly after shooting himself. He has missed his wife very much and believes he will be reunited with her when he dies. He has taken his life to end his emotional pain. [film]

The police tried to intervene but were unsuccessful. He died instantly and painlessly after shooting himself. He believed he would be reunited with his wife when he died. He has taken his life to end his emotional pain.

1. In some parts of Spain, bullfighting still takes place. Sharp darts are fired into the bull’s back, and it is let loose into the streets. The sharp darts draw blood and are painful, hitting key nerves close to the animal’s spine, causing the bull to become frightened and to fight for his survival. Sometimes, people are injured during the bullfights. On this occasion, the bull attacks a woman. As soon as she is attacked, she lays as still a possible to minimise her injuries. She suffers a number of injuries and is rushed to hospital. The doctors are able to stabilise her following major surgery. She survives the incident and now campaigns against bullfighting in Spain. The bull survives and is not called upon in the festival in the following year to fight again. [film]

As soon as the bull had attacked the woman, she had laid as still as possible to minimise her injuries. She suffered a number of injuries and had been rushed to hospital. The doctors were successfully able to stabilise her following major surgery. She survived the incident and she now campaigns against bullfighting in Spain. The bull survived and was not called upon in the festival in the following year to fight again.

1. On a Monday morning at 8:30am, a disastrous pile up is caused on the motorway by a fog zone coming up within a few seconds. As a consequence, 200 cars crash into each other and leave a roadway of 200 yards covered by car wreckage. When people get out of their cars to understand what had happened, a lorry approaches too fast and crashes into the pile up. The truck pushes five cars for a distance of 50 yards compressing them into a small metal ball that is finally stopped by hitting a travel bus. Some of the cars catch fire and burn out before the passengers can free themselves. On the top of the ball of compressed cars, a mother of two is still alive. It is four hours before she is rescued. Her two children are buried underneath the car and she does not know if they are dead or alive. After four hours the woman is successfully rescued. She has a broken leg. She learns that her children had been rescued much earlier. They had been protected by the roof of the car beneath and have no injuries at all. The woman is relieved and is very grateful. [film]

The woman was successfully rescued after four hours. She had a broken leg. She learned that her children had been rescued much earlier. They had been protected by the roof of the car beneath and suffered no injuries at all. The woman was relieved and very grateful.

1. An 18 year old and his friend are celebrating the success of their A-levels. They are driving home from a party at their school in their parents' car. A motorcyclist loses control and collides into them on a bend. He had been drinking. The motorcyclist dies on impact. The boys suffer cuts and bruises and wreck their parents’ car. [film]

The boys survived the accident but the motorcyclist did not. He had been drinking and had collided with them on a bend. He died on impact. The boys had suffered cuts and bruises and had wrecked their parents’ car.

1. A young woman is 6 months pregnant. She and a friend decide to go out for lunch. The friend picks her up and they drive to a restaurant in the next town. The friend drives too fast and the car comes off the road on a bend and crashes into a barrier. The friend survives with injuries. The pregnant woman has to be treated at the accident scene by an emergency doctor. She is airlifted to hospital to undergo surgery. She is in hospital for several weeks after surgery to minimise her movement and to monitor her pregnancy. She and her baby survive and three months after the accident she gives birth to a happy, healthy baby girl. [film]

The young woman was treated at the accident scene by an emergency doctor. She had to be airlifted to hospital to undergo surgery. She was in hospital several weeks after the surgery to minimise her movement and monitor her pregnancy. She and her baby survived and three months after the accident she gave birth to a happy, healthy baby girl.
